# The selenium paradox friend or foe in breast cancer?

**DOI:** 10.1097/MS9.0000000000003464

**Published:** 2025-06-06

**Authors:** Emmanuel Ifeanyi Obeagu

**Affiliations:** Department of Biomedical and Laboratory Sciences, Africa University, Mutare, Zimbabwe

**Keywords:** antioxidant defense, breast cancer, chemoresistance, personalized medicine, selenium

## Abstract

Selenium, an essential trace element, has garnered significant attention for its multifaceted role in cancer biology, particularly in breast cancer. As a component of selenoproteins, selenium contributes to antioxidant defense, immune modulation, and apoptosis regulation, making it a promising candidate for cancer prevention and therapy. Epidemiological and experimental studies suggest that selenium supplementation may reduce oxidative stress, enhance immune surveillance, and suppress tumor initiation in breast cancer. These protective effects underscore selenium’s potential as an ally in combating breast cancer. However, the role of selenium is far from straightforward, as excessive selenium intake or specific genetic contexts can lead to pro-oxidant effects, promoting DNA damage, inflammation, and tumor progression. Moreover, selenium has been implicated in chemoresistance, potentially hindering the efficacy of standard breast cancer treatments. This dual nature, often referred to as the “selenium paradox,” complicates its therapeutic application. The variability in selenium’s impact, influenced by genetic polymorphisms, baseline selenium status, and the tumor microenvironment, underscores the need for a nuanced understanding of its role in breast cancer.

## Introduction

Selenium, a trace element essential for human health, has long been recognized for its antioxidant properties and its role in cancer prevention. As a crucial component of selenoproteins such as glutathione peroxidases (GPx) and thioredoxin reductases (TrxR), selenium contributes to redox balance, immune function, and cellular protection against oxidative stress. Due to these protective attributes, selenium has historically been considered a potential ally in cancer chemoprevention, including for hormone-sensitive malignancies like breast cancer^[1-3]^. Breast cancer remains the most common cancer among women globally and represents a leading cause of cancer-related mortality. Despite advances in screening, diagnosis, and treatment, the complexity and heterogeneity of breast cancer present ongoing challenges to effective management. Researchers have therefore turned to nutritional and micronutrient-based interventions – including selenium supplementation – as adjunct strategies to reduce breast cancer incidence and improve outcomes. However, recent findings have cast doubt on the unidimensional view of selenium as a protective agent^[4-6]^. A growing body of evidence now highlights the paradoxical effects of selenium in the context of breast cancer. While selenium exhibits cytoprotective and anti-tumor activities under certain conditions, high doses or specific selenium compounds may exert pro-oxidant effects or support the survival of malignant cells. Additionally, some clinical and epidemiological studies have shown inconsistent or even adverse associations between selenium status and breast cancer risk, leading to the emergence of what is now known as the “selenium paradox”^[7,8]^.

**HIGHLIGHTS**

- Selenium acts as an antioxidant, reducing oxidative stress and DNA damage linked to breast cancer.
- Dose-dependent effects: low doses are protective, while high doses may cause toxicity or harm.
- Selenium influences cell cycle regulation, apoptosis, and gene expression, aiding in cancer prevention.
- Clinical evidence is inconsistent, with results varying by selenium form, dosage, and population.
- Personalized approaches and further research are needed to optimize selenium’s role in breast cancer care.

This paradox refers to the dualistic nature of selenium’s impact – where its benefits are tightly coupled to narrow dosage windows, chemical form, and individual biological context. For instance, in selenium-deficient populations, supplementation may yield protective effects by correcting redox imbalance. Conversely, in selenium-replete individuals, excess intake may disturb cellular homeostasis or promote oncogenic processes. This dichotomy raises critical questions about the safety and efficacy of selenium-based interventions in breast cancer prevention and treatment^[9-11]^. The chemical form of selenium – whether inorganic (e.g., sodium selenite), organic (e.g., selenomethionine), or metabolite-based (e.g., methylseleninic acid) – further modulates its biological behavior. These forms differ in bioavailability, metabolic pathways, and cellular targets. Notably, selenium’s influence on apoptosis, cell proliferation, angiogenesis, immune response, and epigenetic regulation suggests that its impact is far from uniform and may vary across breast cancer subtypes^[12,13]^. Genetic variability adds another layer to the paradox. Polymorphisms in selenoprotein genes or genes involved in selenium metabolism can influence individual responsiveness to selenium exposure. These genetic differences may partly explain why some patients benefit from selenium supplementation while others experience no benefit – or worse, increased risk. Thus, personalized selenium strategies based on genomic profiling may be necessary to optimize clinical utility and minimize harm^[14,15]^. Beyond its intrinsic effects, selenium’s role in the tumor microenvironment also deserves attention. Selenium has been shown to modulate immune cell function, inflammatory signaling, and the oxidative landscape within tumors. These influences can either support immune-mediated tumor clearance or facilitate immune evasion depending on context. Moreover, selenium’s interaction with standard therapies such as chemotherapy and radiotherapy can alter treatment outcomes, either enhancing efficacy or contributing to resistance^[16]^.

## Aim

This review aims to dissect the dual role of selenium in breast cancer, focusing on its protective and detrimental effects, molecular mechanisms, and therapeutic implications.

### Review methods

To ensure the rigor, transparency, and reproducibility of this narrative review on the paradoxical role of selenium in breast cancer, a structured approach to literature selection and thematic synthesis was employed. A comprehensive literature search was conducted across three major scientific databases: PubMed, Scopus, and Web of Science. These databases were chosen for their extensive indexing of biomedical and multidisciplinary peer-reviewed publications. The search strategy incorporated a combination of Medical Subject Headings (MeSH) and free-text keywords to capture relevant studies. Key search terms included: *“selenium,” “breast cancer,” “selenoproteins,” “oxidative stress,” “antioxidant,” “GPX1,” “SEPP1,” “chemoprevention,”* and *“genetic polymorphism.”* Boolean operators (“AND,” “OR”) were used to refine the search and maximize the retrieval of relevant literature. The inclusion criteria were carefully defined to focus the review on current and high-quality evidence. Articles were eligible for inclusion if they met the following conditions: (1) published between 2000 and 2024; (2) written in English; (3) peer-reviewed original research articles, clinical trials, systematic reviews, or meta-analyses; and (4) studies that directly investigated selenium’s role in breast cancer etiology, prevention, therapy, or molecular mechanisms. Exclusion criteria included non-peer-reviewed sources, editorials, commentaries, conference abstracts without full text, and studies that did not specifically address breast cancer.

Following the initial database search, all retrieved articles were exported into a reference management tool, and duplicate entries were removed. Titles and abstracts were screened independently by two reviewers to assess relevance based on the inclusion criteria. Full texts of potentially eligible articles were then evaluated for their methodological rigor, relevance to the topic, and clarity of reported outcomes. A thematic synthesis approach was applied to analyze and integrate findings from the selected literature. This process involved coding the data into recurring themes, including *selenium’s antioxidant mechanisms, interaction with selenoproteins, dose-dependent effects, genetic variability in selenium response*, and *implications for breast cancer treatment and prevention*. Contradictions and controversies within the literature were critically examined to present a balanced and nuanced perspective.

### Selenium as a friend

Selenium has been widely recognized for its protective role in cancer biology, particularly in its ability to mitigate oxidative stress, modulate immune responses, and regulate apoptosis. These mechanisms collectively contribute to its potential as a valuable ally in breast cancer prevention and therapy.

### Antioxidant defense and oxidative stress mitigation

One of the most well-established protective roles of selenium is its contribution to antioxidant defense. Selenium is a critical component of selenoproteins such as GPx and thioredoxin reductases, which play pivotal roles in neutralizing ROS and preventing oxidative damage. Oxidative stress, a hallmark of cancer, is known to cause DNA damage, lipid peroxidation, and protein oxidation, all of which contribute to tumor initiation and progression. By reducing ROS levels, selenium helps maintain cellular homeostasis and protects DNA integrity, potentially reducing the risk of mutations that drive breast cancer development^[17,18]^.

### Immune system enhancement

Selenium also enhances immune surveillance, which is essential for recognizing and eliminating cancer cells. Studies have shown that selenium supplementation can boost the activity of immune cells such as T-lymphocytes, macrophages, and NK cells. For example, selenium enhances T-cell proliferation and cytokine production, thereby improving adaptive immune responses. It also increases the cytotoxic activity of NK cells, which are crucial for targeting and destroying cancer cells. Through these immunomodulatory effects, selenium may help prevent the establishment and progression of breast cancer^[19]^.

### Regulation of apoptosis

Another key protective mechanism of selenium is its ability to regulate apoptosis, the programmed cell death pathway that serves as a natural defense against cancer. Selenium compounds, such as selenomethionine and selenium nanoparticles, have been shown to induce apoptosis in breast cancer cells through both intrinsic and extrinsic pathways. This is achieved by modulating pro-apoptotic and anti-apoptotic proteins, such as increasing Bax/Bcl-2 ratios, activating caspase cascades, and enhancing the expression of tumor suppressor genes like p53. By selectively inducing apoptosis in cancer cells, selenium may inhibit tumor growth without significantly affecting normal cells^[2]^.

### Anti-inflammatory effects

Chronic inflammation is closely linked to breast cancer progression, and selenium’s anti-inflammatory properties further support its protective role. Selenium inhibits the production of pro-inflammatory cytokines such as interleukin-6 (IL-6) and tumor necrosis factor-alpha (TNF-α), both of which are associated with cancer progression. Additionally, selenium reduces the activity of nuclear factor-kappa B (NF-κB), a key transcription factor involved in inflammation and tumor promotion. By suppressing inflammatory pathways, selenium helps create a microenvironment less conducive to tumor growth and metastasis^[20]^.

### Epigenetic regulation and tumor suppression

Emerging evidence suggests that selenium can modulate epigenetic mechanisms, such as DNA methylation and histone modifications, to exert tumor-suppressive effects. Selenium has been shown to reverse hypermethylation of tumor suppressor genes, restoring their expression and activity. For instance, selenium supplementation can demethylate and reactivate genes that regulate cell cycle arrest and apoptosis, thereby counteracting oncogenic processes. This epigenetic regulation highlights selenium’s potential to influence gene expression profiles associated with breast cancer prevention^[21]^.

### Chemopreventive properties

Selenium’s chemopreventive potential is supported by its ability to reduce the risk of early tumor formation. Preclinical studies have demonstrated that selenium-enriched diets reduce the incidence of mammary tumors in animal models. Selenium’s ability to detoxify carcinogenic compounds and inhibit angiogenesis further reinforces its protective effects. By preventing the formation of new blood vessels that supply nutrients to tumors, selenium disrupts tumor growth and metastasis^[22]^.

### Selenium as a foe

While selenium has demonstrated protective roles in cancer biology, its effects are not universally beneficial. Excessive selenium intake, inappropriate supplementation, and specific molecular contexts can convert selenium from a protector to a promoter of breast cancer progression. This paradoxical behavior, referred to as the “selenium paradox,” underscores its potential as a foe in certain situations^[23]^.

### Pro-oxidant effects at high doses

Although selenium is a key component of the antioxidant defense system, its effects are highly dose-dependent. At high concentrations, selenium can act as a pro-oxidant, leading to the generation of ROS rather than their neutralization. Elevated ROS levels can damage cellular components, including DNA, lipids, and proteins, and promote mutagenesis. This oxidative stress creates a favorable environment for tumor initiation and progression, particularly in cells already vulnerable to genetic instability. Such findings highlight the narrow therapeutic window of selenium, where both deficiency and excess can pose risks^[24]^.

### Promotion of tumor growth in certain contexts

Evidence suggests that selenium may promote tumor growth and survival in specific cellular and molecular contexts. For example, selenium compounds have been shown to enhance the expression of survival pathways, such as the phosphoinositide 3-kinase (PI3K)/Akt signaling cascade, which is frequently dysregulated in breast cancer. Activation of these pathways can suppress apoptosis, promote cell proliferation, and enhance cancer cell survival. This paradoxical effect may explain why selenium supplementation has been associated with increased cancer risk in some studies, particularly in individuals with preexisting malignancies or high baseline selenium levels^[25]^.

### Chemoresistance and therapeutic interference

Selenium’s interaction with standard breast cancer therapies presents another challenge. While selenium’s antioxidant properties can protect normal tissues from the toxic effects of chemotherapy and radiotherapy, they may also shield cancer cells from these treatments. By reducing oxidative stress and DNA damage, selenium can decrease the efficacy of therapies that rely on generating reactive oxygen species (ROS) to kill cancer cells. This protective effect, though beneficial for normal tissues, may inadvertently contribute to chemoresistance, reducing treatment outcomes in breast cancer patients^[26]^.

### Genetic variations and differential responses

Polymorphisms in selenoprotein genes, such as GPx1 and selenoprotein P (SELENOP), can influence how selenium impacts breast cancer. Certain genetic variants may reduce the efficiency of selenium utilization, altering its protective effects and potentially exacerbating its detrimental ones. These genetic factors also contribute to interindividual variability in selenium’s impact, complicating the development of universal supplementation guidelines. For individuals with specific polymorphisms, selenium supplementation may lead to unexpected outcomes, including increased cancer risk or progression^[27]^.

### Tumor microenvironment modulation

Selenium’s influence on the tumor microenvironment can have contradictory effects. While selenium has been shown to enhance immune surveillance in some studies, it can also promote angiogenesis and support the survival of cancer-associated fibroblasts in others. These stromal components play critical roles in breast cancer progression, providing the necessary support for tumor growth and metastasis. Under certain conditions, selenium may inadvertently enhance these pro-tumorigenic processes, contributing to disease progression^[28]^.

### Over-supplementation risks in high-selenium populations

Populations with high baseline selenium levels, such as those in regions with selenium-rich soils or individuals consuming selenium-enriched diets, are particularly vulnerable to the adverse effects of over-supplementation. In such cases, additional selenium intake can disrupt homeostasis, leading to toxicity and an increased risk of chronic diseases, including certain cancers. Clinical trials, such as SELECT, have highlighted these risks, showing a potential increase in cancer incidence with excessive selenium supplementation in some groups^[29]^.

### Inflammatory and epigenetic dysregulation

Although selenium has anti-inflammatory properties, excessive levels can exacerbate inflammation in certain contexts. High selenium concentrations have been shown to activate NF-κB and other inflammatory pathways, which can promote tumor growth. Additionally, selenium’s impact on epigenetic regulation may have unintended consequences. For example, while selenium can reverse hypermethylation of tumor suppressor genes, it may also alter the expression of oncogenes or other regulatory pathways, inadvertently promoting malignancy^[30]^.

### Potential cytotoxicity and off-target effects

At elevated doses, selenium compounds, such as selenite, can become cytotoxic, damaging not only cancer cells but also normal tissues. This nonspecific toxicity poses challenges for therapeutic applications, particularly in individuals with comorbid conditions or compromised health. Off-target effects of selenium supplementation, including gastrointestinal disturbances, liver toxicity, and endocrine dysregulation, further limit its utility as a universally beneficial agent^[31]^.

### Conflicting epidemiological evidence

Epidemiological studies examining the relationship between selenium and breast cancer risk have yielded conflicting results, reflecting the complexity of selenium’s role. While selenium deficiency has been linked to increased cancer risk in some populations, excessive selenium levels have been associated with higher cancer incidence in others. These discrepancies highlight the importance of context, including baseline selenium status, genetic factors, and environmental influences, in determining selenium’s impact^[32]^.

### Need for precision supplementation

Given selenium’s dual nature, a one-size-fits-all approach to supplementation is unlikely to succeed. Personalized strategies that consider an individual’s genetic profile, baseline selenium levels, and breast cancer subtype are essential for optimizing its use. Without these considerations, selenium supplementation carries significant risks, including exacerbating disease progression, promoting chemoresistance, and causing systemic toxicity^[33]^.

### A deeper insight into molecular pathways affected by selenium in breast cancer

Selenium exerts a wide range of biological effects in breast cancer, largely mediated through its incorporation into selenoproteins and its influence on key molecular signaling pathways. At the forefront of selenium’s molecular action is its role in redox homeostasis. Selenium is an essential component of glutathione peroxidases (GPx) and thioredoxin reductases (TrxR), enzymes that neutralize reactive oxygen species (ROS) and protect cells from oxidative stress. In normal and pre-malignant breast epithelial cells, selenium supports genomic stability by minimizing oxidative DNA damage. However, in established breast cancer cells, particularly under high-dose selenium exposure, this antioxidant activity may shift toward a pro-oxidant effect, selectively inducing oxidative stress within tumor cells. This oxidative imbalance can trigger apoptosis, autophagy, or, in some cases, promote genetic instability and tumor adaptation^[34]^. Selenium also modulates critical apoptotic and cell survival pathways. Methylseleninic acid (MSA), a selenium metabolite, has been shown to activate caspase cascades – particularly caspase-3 and -9 – through the intrinsic mitochondrial pathway. This involves disruption of the mitochondrial membrane potential and release of cytochrome c, events that are often suppressed in malignant cells. Additionally, selenium downregulates anti-apoptotic proteins such as Bcl-2 while upregulating pro-apoptotic markers like Bax, thereby tipping the balance toward programmed cell death in breast cancer cells^[5]^.

The p53 tumor suppressor pathway also appears to be influenced by selenium. Selenium compounds have been reported to enhance the expression and activity of p53, leading to cell cycle arrest and apoptosis in breast cancer models. This p53 activation may be particularly relevant in triple-negative breast cancers (TNBCs), where therapeutic options are limited, and restoring tumor suppressor pathways could have significant clinical benefits ^[35]^. Furthermore, selenium impacts cell proliferation through modulation of key oncogenic signaling cascades such as the PI3K/Akt/mTOR and MAPK/ERK pathways. Inhibition of these pathways by selenium compounds results in reduced cell proliferation, migration, and invasion. For instance, MSA has been demonstrated to suppress the phosphorylation of Akt and ERK1/2, thereby dampening downstream signals involved in cell growth and survival^[36]^. Angiogenesis, the process by which tumors establish their blood supply, is also targeted by selenium. Studies have shown that selenium suppresses the expression of vascular endothelial growth factor (VEGF) and hypoxia-inducible factor-1 alpha (HIF-1α), key mediators of angiogenesis. By impairing the vascularization of tumors, selenium restricts nutrient delivery and oxygen supply, contributing to tumor regression^[22]^. Moreover, selenium may modulate epigenetic regulators and transcription factors. Evidence suggests that selenium influences DNA methylation patterns and histone modifications, potentially altering the expression of genes involved in tumor suppression and immune regulation. Additionally, selenium has been reported to affect nuclear factor-kappa B (NF-κB), a transcription factor involved in inflammation and cancer progression. Selenium-induced inhibition of NF-κB signaling may reduce inflammatory cytokine production and tumor-promoting inflammation within the tumor microenvironment^[32]^. Selenium’s immunomodulatory effects also intersect with molecular pathways. By enhancing the activity of natural killer (NK) cells and cytotoxic T lymphocytes (CTLs), selenium may reinforce the immune system’s capacity to recognize and eliminate breast cancer cells. This is particularly significant in the context of immunoediting, where tumors evolve to evade immune surveillance^[37]^.

### Tumor microenvironment modulation

Selenium’s influence extends beyond cancer cells to the tumor microenvironment (TME), which plays a critical role in breast cancer progression. Selenium can modulate immune cell activity, angiogenesis, and extracellular matrix remodeling within the TME. While selenium’s ability to enhance immune surveillance and inhibit angiogenesis can suppress tumor growth, it may also support cancer-associated fibroblasts and endothelial cells in certain contexts, potentially promoting tumor survival and metastasis.

### Hormonal interactions in breast cancer

As an estrogen-sensitive cancer, breast cancer is influenced by hormonal pathways that interact with selenium metabolism. Selenium has been shown to modulate estrogen receptor signaling, which plays a key role in hormone-responsive breast cancer. While selenium can inhibit estrogen-driven tumor growth in some studies, it may also enhance the survival of hormone-resistant cancer cells by modulating alternative signaling pathways^[37]^.

### Chemoresistance and radiotherapy interference

Selenium’s antioxidant properties, while protective for normal cells, may interfere with cancer therapies that rely on ROS generation. For example, selenium can reduce the oxidative damage induced by chemotherapeutic agents, thereby decreasing their efficacy in killing cancer cells. Similarly, selenium’s radioprotective effects may shield cancer cells from the DNA-damaging effects of radiotherapy, contributing to treatment resistance.

### Role of selenium transport and metabolism

Selenium’s bioavailability and distribution are tightly regulated by transport proteins, such as SELENOP, and metabolic enzymes, such as selenocysteine lyase. Dysregulation of these processes can alter selenium’s effects, contributing to its paradoxical behavior. For instance, increased expression of SELENOP in the tumor microenvironment may enhance selenium delivery to cancer cells, supporting their growth and survival^[38]^.

### Selenium in therapy

Selenium’s potential therapeutic applications in breast cancer have garnered considerable attention due to its antioxidant, anti-inflammatory, and immune-modulating properties. However, its dual role as both a protective agent and a potential promoter of cancer progression necessitates careful consideration of its dosage, chemical form, and patient-specific factors. Current research focuses on optimizing selenium-based therapies while mitigating its paradoxical effects.

### Selenium as an adjunct to chemotherapy and radiotherapy

Selenium has shown promise as an adjunct to conventional cancer therapies. Its antioxidant properties can protect normal cells from oxidative damage caused by chemotherapy and radiotherapy, potentially reducing treatment-related side effects such as fatigue, mucositis, and neuropathy. For example, selenium supplementation has been associated with decreased cardiotoxicity in patients undergoing anthracycline-based chemotherapy. However, selenium’s protective effects may extend to cancer cells, reducing the efficacy of therapies that rely on oxidative stress to induce cell death. This highlights the need for precise timing and dosing of selenium supplementation to selectively benefit normal tissues without compromising cancer treatment outcomes. Some studies suggest that selenium’s organic forms, such as methylselenocysteine, may strike a better balance between protective and anti-cancer effects compared to inorganic forms like selenite^[39]^.

### Selenium and hormonal therapies in breast cancer

Given the hormone-sensitive nature of many breast cancers, selenium’s interactions with estrogen receptor (ER) signaling have been explored. Selenium has been shown to inhibit the growth of ER-positive breast cancer cells by modulating estrogen signaling and inducing apoptosis. Additionally, selenium may enhance the efficacy of selective estrogen receptor modulators (SERMs), such as tamoxifen, by synergistically inhibiting cancer cell proliferation. For ER-negative or triple-negative breast cancers, which are more aggressive and lack targeted hormonal therapies, selenium may still offer therapeutic benefits. Studies indicate that selenium compounds can inhibit tumor growth in these subtypes by modulating signaling pathways, such as NF-κB and PI3K/Akt, and promoting apoptosis. These findings support the potential role of selenium as a complementary strategy for diverse breast cancer subtypes^[40]^.

### Selenium nanoparticles

Selenium nanoparticles (SeNPs) represent an innovative approach to harness selenium’s therapeutic potential while minimizing its toxicity. These nanoscale formulations exhibit enhanced bioavailability, targeted delivery, and controlled release, making them a promising option for breast cancer therapy. SeNPs have demonstrated selective cytotoxicity toward cancer cells while sparing normal cells. This selective action is attributed to their ability to induce oxidative stress and apoptosis in cancer cells through mechanisms involving mitochondrial dysfunction, DNA damage, and activation of caspase cascades. Moreover, SeNPs can be functionalized with targeting ligands, such as antibodies or peptides, to improve their specificity for breast cancer cells, thereby enhancing therapeutic efficacy and reducing off-target effects^[41]^.

### Immunomodulatory effects of selenium in therapy

Selenium’s role in modulating the immune system offers another therapeutic dimension in breast cancer. By enhancing the activity of natural killer (NK) cells, cytotoxic T lymphocytes, and macrophages, selenium may improve immune surveillance and anti-tumor immunity. Selenium has also been shown to reduce the immunosuppressive activity of regulatory T cells (Tregs) and myeloid-derived suppressor cells (MDSCs), which are often elevated in the tumor microenvironment and contribute to immune evasion. Furthermore, selenium supplementation has been investigated in combination with immune checkpoint inhibitors, such as anti-PD-1 and anti-CTLA-4 therapies. Preliminary studies suggest that selenium may enhance the efficacy of these immunotherapies by modulating the tumor microenvironment and improving immune cell infiltration^[42]^.

### Selenium as a preventive agent in high-risk populations

In addition to its therapeutic applications, selenium has been evaluated as a preventive agent in individuals at high risk for breast cancer, such as those with a family history or genetic predisposition. Selenium’s ability to reduce oxidative stress, modulate inflammation, and enhance DNA repair mechanisms makes it a candidate for chemoprevention. However, large-scale clinical trials have yielded mixed results regarding selenium’s preventive efficacy. While some studies report a reduction in breast cancer risk with selenium supplementation, others show no significant benefit or even an increased risk in specific populations, such as those with adequate baseline selenium levels. These findings underscore the importance of personalized approaches to selenium supplementation, considering factors such as baseline selenium status, genetic polymorphisms, and environmental exposures^[43]^.

### Promises of selenium in breast cancer

The promises of selenium in breast cancer prevention and management are centered around its biological properties and potential therapeutic benefits. Here are the key areas where selenium has shown promise:

### Antioxidant protection

Selenium is a key component of selenoproteins, such as glutathione peroxidases (GPX), which play a vital role in protecting cells from oxidative damage. Since oxidative stress contributes to DNA mutations and cancer initiation, selenium’s antioxidant properties could help reduce the risk of breast cancer development by neutralizing free radicals and preventing cellular damage^[36]^.

### Immune system enhancement

Selenium has been shown to modulate immune function, potentially enhancing the body’s ability to recognize and destroy abnormal cells, including cancerous ones. Selenium influences T-cell function and cytokine production, which can boost immune surveillance and help the body fight off cancer cells before they become problematic^[22]^.

### Regulation of the cell cycle and apoptosis

Selenium plays a role in the regulation of genes involved in the cell cycle and apoptosis (programmed cell death). It can enhance the activity of tumor suppressor genes, such as p53, which promote apoptosis in damaged or cancerous cells. By encouraging the death of abnormal cells, selenium may help prevent the proliferation of breast cancer cells^[32]^.

### Epigenetic modulation

Selenium has the potential to influence epigenetic mechanisms, such as DNA methylation, histone modification, and microRNA expression. These processes control gene expression without altering the DNA sequence, and selenium’s ability to modulate these changes may be crucial in preventing breast cancer or reversing early-stage carcinogenesis^[37]^.

### Synergistic effects with chemotherapy

Some studies suggest that selenium could enhance the efficacy of conventional chemotherapy. By promoting oxidative stress specifically in cancer cells, selenium may sensitize tumor cells to chemotherapy agents, making them more effective. Additionally, selenium may help reduce the side effects of chemotherapy, offering a dual benefit in treatment^[38]^.

### Reduction in inflammation

Chronic inflammation is a known risk factor for breast cancer development. Selenium’s anti-inflammatory effects can reduce the production of pro-inflammatory cytokines and other mediators that contribute to cancer initiation and progression. This anti-inflammatory action might help reduce the risk of inflammation-induced tumorigenesis^[39]^.

### Protection against hormone-driven cancers

Breast cancer can be hormonally driven, particularly in estrogen receptor-positive subtypes. Some studies have suggested that selenium may help modulate estrogen metabolism, thereby reducing the availability of estrogen to promote tumor growth. This is particularly important for understanding how selenium might play a role in preventing hormone-dependent breast cancers^[40]^.

### Improved breast cancer survival

Emerging research indicates that adequate selenium levels may be associated with improved survival rates in breast cancer patients. Studies have shown that selenium supplementation may reduce recurrence and metastasis in some breast cancer patients, potentially contributing to better long-term outcomes^[41]^.

## Challenges

While selenium holds promise in breast cancer prevention and treatment, several challenges complicate its effective use and integration into clinical practice. Here are some of the primary challenges associated with selenium in breast cancer:

### Dose-dependent effects

Selenium’s effects are highly dose-dependent, and while low to moderate levels may provide protective benefits, high doses can have toxic effects. The narrow therapeutic window between the beneficial and harmful doses complicates its use as a preventive or therapeutic agent. Excessive selenium intake may increase the risk of adverse outcomes, such as selenium toxicity, which can lead to symptoms like gastrointestinal distress, hair loss, and even organ damage^[42]^.

### Selenium form matters

The bioavailability and efficacy of selenium can vary depending on its chemical form. Organic forms, like selenomethionine, are generally better absorbed and utilized by the body, whereas inorganic forms, like sodium selenite, may be less effective or even harmful at higher doses. This variation in selenium forms further complicates supplementation strategies and the interpretation of clinical trials^[43]^.

### Genetic variability

Individuals have different genetic makeups that can influence how their bodies process and respond to selenium. Genetic variations in selenoprotein genes, such as GPX1, can affect the antioxidant properties of selenium and its impact on cancer prevention. Some individuals may have a higher susceptibility to selenium’s toxic effects, while others may benefit more from its protective actions. Personal genetic profiles need to be considered when evaluating selenium’s potential in breast cancer treatment.

### Inconsistent clinical evidence

Clinical studies and trials investigating selenium’s role in breast cancer prevention and treatment have produced mixed results. Some studies show promising effects of selenium in reducing breast cancer risk, improving survival, or enhancing chemotherapy outcomes, while others show no significant benefits. The inconsistency in findings can be attributed to differences in study design, patient populations, selenium dosages, and the forms of selenium used. These inconsistencies make it difficult to form definitive conclusions about selenium’s role in breast cancer^[34]^.

### Lack of standardized guidelines

There are no universally accepted guidelines for selenium supplementation in breast cancer prevention or treatment. Recommendations regarding optimal dosages, duration of supplementation, and the ideal form of selenium remain unclear. Without standardization, healthcare providers may be uncertain about how to incorporate selenium into breast cancer care, resulting in inconsistent practices^[5]^.

### Potential for pro-carcinogenic effects

As discussed earlier, selenium may not always act as a protective agent. In some circumstances, particularly at high doses or with certain forms of selenium, it may contribute to cancer progression rather than prevention. High selenium levels can increase the production of reactive oxygen species (ROS) and lead to oxidative damage, genomic instability, and tumor cell survival. This paradoxical effect complicates its use in breast cancer therapy^[35]^.

### Lack of long-term safety data

While short-term selenium supplementation appears to be relatively safe, the long-term effects of selenium supplementation, particularly in high-risk breast cancer populations, are not well understood. More research is needed to assess the safety of prolonged selenium use, especially for individuals already undergoing cancer treatments like chemotherapy or radiation, which may interact with selenium^[36]^.

### Bioavailability and dietary factors

The bioavailability of selenium is influenced by various factors, including diet and geographical location. For example, selenium levels in soil and food can vary widely, which means individuals in selenium-deficient regions might benefit more from supplementation compared to those living in areas with adequate selenium in the food supply. This variability further complicates efforts to determine the optimal selenium intake for breast cancer prevention on a global scale^[22]^.

### Potential drug interactions

Selenium may interact with certain medications, including chemotherapy drugs, altering their effectiveness or increasing side effects. For instance, some studies suggest that selenium could enhance the effects of chemotherapy, while others indicate it might interfere with the action of specific drugs. The risk of drug interactions underscores the need for careful monitoring when combining selenium with other cancer therapies.^[32,37-39]^

### Individualized approaches needed

Given the complexity of selenium’s effects and the various factors influencing its activity, an individualized approach to selenium supplementation in breast cancer prevention and therapy is essential. Personalizing treatment based on factors such as genetic predisposition, current selenium levels, and cancer subtype will be crucial to maximize its potential benefits and minimize risks.^[40-43]^

### Future directions

The evolving understanding of selenium’s paradoxical role in breast cancer underscores the pressing need for a shift from generalized nutritional recommendations to more precise, individualized approaches. As emerging evidence increasingly highlights the differential effects of selenium based on dosage, chemical form, and host factors, the concept of personalized medicine becomes central to optimizing selenium’s role in cancer prevention and therapy^[44]^. One of the most promising future directions involves the integration of genomic and metabolomic profiling to tailor selenium supplementation. Genetic polymorphisms in selenoprotein-encoding genes – such as GPX1, SEPP1, and SELENOP – significantly influence selenium metabolism, selenoprotein expression, and the body’s overall response to selenium. For instance, individuals with certain variants of *GPX1* (e.g., Pro198Leu) may exhibit altered enzyme activity and reduced antioxidant defense, potentially modifying their susceptibility to breast cancer in response to selenium intake. Future clinical studies should incorporate such genetic markers to stratify patients into selenium-responsive or selenium-resistant categories^[5,29,45]^. Additionally, personalized selenium dosing strategies must account for baseline selenium status, which varies significantly across global populations due to dietary intake, soil selenium content, and supplementation practices. Blanket recommendations for selenium supplementation may therefore be inappropriate or even harmful in selenium-replete populations, potentially tipping the redox balance toward pro-oxidative states that favor tumorigenesis. Monitoring serum selenium levels, as well as the activity of selenium-dependent enzymes, could inform safer and more effective therapeutic thresholds^[46,47]^.

Furthermore, the form of selenium administered should not be viewed as interchangeable. Preclinical data demonstrate that organic selenium compounds (such as selenomethionine) and inorganic forms (such as sodium selenite) differ markedly in their bioactivity and toxicity profiles. Methylseleninic acid (MSA) and other metabolite-based forms appear more promising for therapeutic use due to their selective pro-apoptotic effects on breast cancer cells without exerting significant toxicity on normal tissues. Future clinical trials should prioritize these metabolically active compounds and investigate optimal formulations, delivery systems, and treatment windows^[48,49]^. There is also an urgent need for more rigorous translational research to bridge laboratory findings with clinical applications. Many of the mechanistic insights into selenium’s anticancer actions – such as modulation of PI3K/Akt, NF-κB, and p53 pathways – remain largely confined to in vitro or animal models. Human studies exploring how these pathways are affected in real-world patient populations will be essential. Incorporating selenium biomarker analysis into ongoing breast cancer trials could yield critical data on selenium-pathway interactions and their impact on therapeutic outcomes^[50]^. In the context of combination therapies, selenium’s potential role as a chemosensitizer or radioprotective agent should be explored with caution and scientific rigor. Selenium has demonstrated both synergistic and antagonistic interactions with standard treatments, depending on timing, dose, and tumor subtype. Identifying optimal co-administration protocols may enhance treatment efficacy while minimizing side effects. However, such strategies must be underpinned by comprehensive pharmacokinetic and pharmacodynamic evaluations^[51]^. Advancing population-based research in underrepresented regions with diverse genetic backgrounds and varying selenium statuses is critical. Many existing studies are skewed toward Western populations, limiting global applicability. Collaborative, multi-center trials that consider geographical and ethnic variability will offer broader insights into selenium’s effects and inform equitable clinical guidelines^[52]^.

## Conclusion

The role of selenium in breast cancer prevention and management presents both promising possibilities and significant challenges. As an essential trace element with antioxidant properties, selenium has the potential to reduce oxidative stress, modulate immune responses, regulate the cell cycle and apoptosis, and even enhance the effectiveness of chemotherapy. Its multifaceted mechanisms suggest that it could play an important role in reducing breast cancer risk, improving survival outcomes, and potentially supporting cancer therapies. However, the promise of selenium is tempered by the complexities surrounding its use. The narrow therapeutic window between beneficial and toxic doses, the varying efficacy of different selenium forms, genetic variability in response, and inconsistent clinical evidence present significant barriers to its widespread use. Additionally, concerns about potential pro-carcinogenic effects, long-term safety, and drug interactions further complicate its role in cancer care.

## Data Availability

Not applicable.
